# Risk factors for early mortality in elderly patients with unstable isolated C2 odontoid fracture treated with halo-vest or surgery

**DOI:** 10.1038/s41598-023-45180-6

**Published:** 2023-10-20

**Authors:** Akira Honda, Yoichi Iizuka, Nobuaki Michihata, Kojiro Morita, Tokue Mieda, Eiji Takasawa, Sho Ishiwata, Yohei Kakuta, Yusuke Tomomatsu, Shunsuke Ito, Kazuhiro Inomata, Hiroki Matsui, Kiyohide Fushimi, Hideo Yasunaga, Hirotaka Chikuda

**Affiliations:** 1 Department of Orthopaedic Surgery, Gunma University Graduate School of Medicine, 3-39-22, Showa, Maebashi, Gunma 371-8511 Japan; 2 Cancer Prevention Center, Chiba Cancer Center Research Institute, 666-2, Nitona, Chuo-ku, Chiba, 260-8717 Japan; 3 Global Nursing Research Center, Graduate School of Medicine, University of Tokyo, 7-3-1, Hongo, Bunkyo-ku, Tokyo, 113-0033 Japan; 4 Department of Clinical Epidemiology and Health Economics, School of Public Health, The University of Tokyo, 7-3-1, Hongo, Bunkyo-ku, Tokyo, 113-0033 Japan; 5 Department of Health Policy and Informatics, Tokyo Medical and Dental University Graduate School of Medicine, Tokyo, 45-5-1 Yushima, Bunkyo-ku, Tokyo, 113-8510 Japan

**Keywords:** Medical research, Outcomes research

## Abstract

The aim of this study was to compare in-hospital mortality of three procedures –halo-vest immobilization, anterior spinal fixation (ASF), and posterior spinal fixation (PSF)– in the treatment of elderly patients with isolated C2 odontoid fracture. We extracted data for elderly patients who were admitted with C2 odontoid fracture and treated with at least one of the three procedures (halo-vest immobilization, ASF, or PSF) during hospitalization. We conducted a generalized propensity score-based matching weight analysis to compare in-hospital mortality among the three procedures. We further investigated independent risk factors for in-hospital death. The study involved 891 patients (halo-vest, n = 463; ASF, n = 74; and PSF, n = 354) with a mean age of 78 years. In-hospital death occurred in 45 (5.1%) patients. Treatment type was not significantly associated with in-hospital mortality. Male sex (odds ratio 2.98; 95% confidence interval 1.32–6.73; *p* = 0.009) and a Charlson comorbidity index of ≥ 3 (odds ratio 9.18; 95% confidence interval 3.25–25.92; *p* < 0.001) were independent risk factors for in-hospital mortality. In conclusion, treatment type was not significantly associated with in-hospital mortality in elderly patients with isolated C2 odontoid fracture. Halo-vest immobilization can help to avoid adverse events in patients with C2 odontoid fracture who are considered less suitable for surgical treatment.

## Introduction

The incidence of C2 odontoid fracture in elderly patients has increased during the past two decades because of expansion of the geriatric population worldwide^1^. In elderly patients, C2 odontoid fracture mostly results from low-energy impacts such as falls^2^. With this increase in the number of elderly patients sustaining C2 odontoid fracture, the number of conservative treatments has increased by two to three times in the last decade^3^. Because most elderly patients have comorbidities and high baseline mortality, optimal management of odontoid fracture has long been a major concern.

We previously reported that most patients with C2 odontoid fracture were elderly and treated conservatively without halo-vest^4^. However, when the fracture is unstable and conservative treatment with a cervical collar is insufficient, we should consider external immobilization using a halo-vest or surgical treatment involving anterior spinal fixation (ASF) or posterior spinal fixation (PSF)^5^. Although a halo-vest is widely used for conservative treatment, several studies showed that halo-vest immobilization had worse survival outcomes than surgery^6^. The mortality rate of elderly people treated with halo-vest ranged from 16 to 42%; thus, halo-vest was considered less suitable for elderly patients than young patients^7^. In contrast, several other studies showed no association between the treatment type and clinical outcome^2,8^. This controversy makes it difficult for clinicians to select the optimal treatment for patients with unstable odontoid fracture, especially in elderly patients who are likely to have several comorbidities.

In the present study, we conducted a generalized propensity score-based analysis to compare clinical outcomes among halo-vest immobilization, ASF, and PSF in elderly patients with unstable isolated C2 odontoid fracture.

## Materials and methods

### Data source

Inpatient data were extracted from the Japanese Diagnosis Procedure Combination database, a national database containing administrative claims and discharge data^9^. All academic hospitals are obliged to participate in the database, and more than 1000 community hospitals voluntarily contribute to the database. Overall, the database provides data for approximately 50% of all acute-care inpatients in Japan. The database contains the following information: encrypted unique identifiers; age and sex; body weight and height; admission and discharge dates; diagnoses coded according to the International Classification of Diseases (ICD), 10th revision; surgical and nonsurgical procedures coded according to Japanese original codes (K codes); drugs prescribed; and discharge status. A previous study showed that the validity of diagnoses and procedure records in the database was high (sensitivity and specificity of primary diagnoses: 78.9% and 93.2%, respectively)^10^. The database clearly differentiates between comorbidities that were already present at admission and complications that occurred after admission, and many studies using the database have been reported elsewhere^4,11,12^.

This study was approved by the Institutional Review Board of The University of Tokyo [approval number: 3501-(3) (December 25th, 2017)]. The requirement for informed consent was waived by the Ethics committee of The University of Tokyo because of the anonymous nature of the data. All study were performed in accordance with relevant guidelines and regulations.

### Patient selection

From July 2010 to March 2017, we screened all patients who were admitted with C2 fracture (ICD-10 code: S12.1) and further identified odontoid fracture using Japanese disease codes. The inclusion criteria were age of ≥ 65 years and admission for treatment of odontoid fracture by at least one of three procedures (halo-vest immobilization (K1444), ASF(K142-1), or PSF (K142-2)) during hospitalization. We excluded patients with multiple fractures (any fractures other than odontoid fractures), with severe consciousness disturbance at admission, who underwent combined surgery (both ASF and PSF), or who died within 2 days of admission. The patients who were treated with halo-vest before or after ASF or PSF were included in the surgery group.

### Covariates and outcomes

We compared the three procedures (halo-vest immobilization, ASF, and PSF) using the following covariates at admission: age; sex; body mass index (BMI) (kg/m^2^); smoking status; academic hospitals; emergency admission; ambulance use; primarily admitted to intensive care unit; oxygenation, hemodialysis, or renal catheter use on admission; pre-existing comorbidities such as diabetes mellitus (E10–E14), hypertension (I10–I15), or chronic lung disease (J40–J47); history of cerebrovascular disease (I60–I69), cardiac disease (I20–I25, I30–I52), hepatic cirrhosis (K74), or dementia (F00–F03); Japan Coma Scale score on admission, which is correlated with the Glasgow Coma Scale score^13^; Charlson comorbidity index (CCI)^14^; and Barthel index^15^. Use of navigation (K9391) was identified among the surgical groups. We categorized eligible patients into two age groups: 65 to 79 years and ≥ 80 years. BMI was categorized into underweight (< 18.5 kg/m^2^), normal weight (18.5–24.9 kg/m^2^), overweight (25.0–29.9 kg/m^2^), obesity (≥ 30.0 kg/m^2^), and missing according to the World Health Organization definition. Smoking status was categorized into nonsmoking, smoking, and missing.

The primary endpoint was overall in-hospital mortality. The secondary endpoints were at least one complication after admission, post-treatment length of stay (PLOS), and total hospitalization cost in US dollars (USD). We identified complications after admission from the diagnoses recorded after admission using the following ICD-10 codes and defined at least one complication as at least one of the following complications during hospitalization: sepsis (A40–A41), pulmonary embolism (I26), respiratory complications [pneumonia (J12–J18, J69), respiratory failure (J96), respiratory disorders (J95)], acute coronary syndrome (I21–I24), heart failure (I50), stroke (I60–I64), urinary tract infection (N30, N34, N36–N37, N39), and renal failure (N17–N19). PLOS was defined as the length of stay from the day treated with halo-vest, ASF, or PSF to discharge (or death). Total hospitalization cost includes item-by-item prices for surgical, pharmaceutical, laboratory, nursing care, and other inpatient services, that are offered by universal health care in Japan. The currency exchange rate was set at 100 Japanese yen per USD to account for the average rate of the study period.

### Statistical analysis

We used a propensity score-based method to account for differences in observed factors that might affect either the treatment assignment or outcome^16^. The propensity score was defined as the probability of a patient undergoing halo-vest immobilization, ASF, or PSF based on the patient’s baseline covariates. Covariate selection was prespecified by using both potential confounding factors and variables that can serve as proxies for unknown or unmeasured confounding variables. The propensity score was estimated using a multinomial logistic model with the procedure received as the dependent variable and the following baseline factors as independent variables^17^: age; sex; BMI category; smoking status; ambulance use; emergency admission; admission to intensive care unit before treatment; oxygenation therapy before treatment; use of urinary catheter; pre-existence of diabetes mellitus, hypertension, or chronic lung disease; history of cerebrovascular disease, cardiac disease, hepatic disease, dementia, or osteoporosis; at least one comorbidity; Japan Coma Scale score category; Barthel index; and CCI category on admission.

To balance the patients’ baseline characteristics among the three procedures, a matching weight approach was applied^18^. Matching weights is recommended for comparing outcomes across multiple treatment groups when the covariates’ overlaps are relatively limited, outcomes are rare, or exposure distributions are unequal^19^. Each patient was weighted by the inverse probability with the lower propensity score of the three procedures as the numerator^19^. The patients would receive each of the treatments among halo-vest immobilization, ASF, or PSF, allowing average treatment effects to be estimated. Baseline covariate balance was checked after weighting, using a *p* value of > 0.05 calculated by analysis of variance or the chi-squared test among the three treatments.

We compared the following outcomes among the three groups (halo-vest immobilization, ASF, and PSF) using analysis of variance and the chi-square test in the matching weighted cohort: overall in-hospital death, complications after admission, PLOS, total hospitalization costs, and Barthel index at discharge. We further conducted logistic regression analyses to estimate the odds ratios (ORs) and 95% confidence intervals (CIs) for overall in-hospital death and at least one complication after admission. We also conducted a linear regression analysis to estimate the regression coefficient and 95% CI for the PLOS. Moreover, we conducted a multivariable logistic regression analysis with adjustment for age, sex, BMI category, smoking status, and CCI category in the non-weighted and weighted cohorts to identify risk factors for in-hospital death. The following sensitivity analyses were undertaken to assess the robustness of the results. We combined the ASF and PSF groups as the surgery group and compared halo-vest immobilization with the surgery group using propensity score-matching analysis and matching weight analysis to balance the baseline variables.

Statistical analyses were performed using Stata/MP version 15 software (StataCorp, College Station, TX, USA). A two-tailed significance level of *p* < 0.05 and 95% CIs were used in the analyses.

### Ethical approval

The study design was approved by the Institutional Review Board of The University of Tokyo.

### Consent to participate

The requirement for informed consent was waived because of the anonymous nature of the data.

## Results

**Figure 1:**
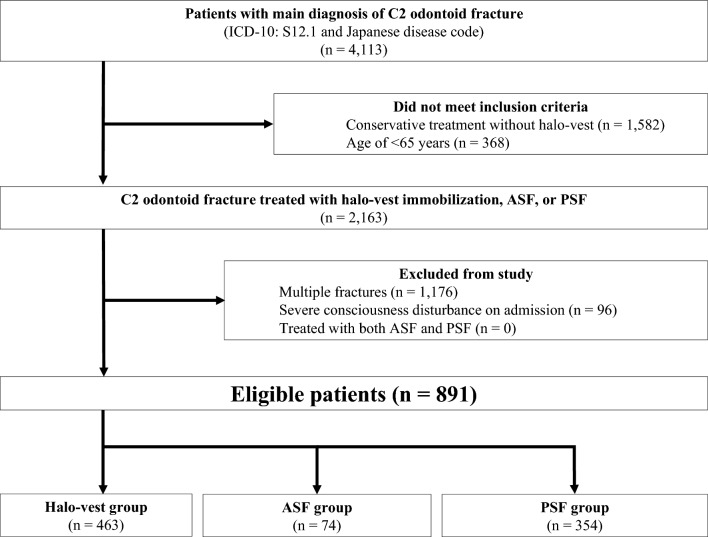
Flow chart of patients. We screened all patients who were admitted with C2 fracture (ICD-10 code: S12.1) and further identified odontoid fracture with the Japanese disease code. After excluding patients who were treated conservatively without halo-vest and who were aged < 65 years, we further excluded patients who had severe consciousness disturbance, had multiple injuries, and died within 2 days after admission to exclude critically ill patients. Finally, 891 patients with isolated C2 odontoid fracture were identified (halo-vest, n = 463; ASF, n = 74; PSF, n = 354). ICD-10, International Classification of Diseases, 10th revision; ASF, anterior spinal fixation; PSF, posterior spinal fixation.

We finally included 891 patients (halo-vest, n = 463; ASF, n = 74; and PSF, n = 354) with isolated C2 odontoid fracture (Fig. 1). More than half of the patients underwent halo-vest immobilization. Table 1 shows the patients’ baseline characteristics before and after matching weight. Overall, 366 patients (41%) were male, and their mean age was 78 ± 7.5 years. Navigation was used in 115 patients among the surgical groups (ASF: 8/74, 11%; PSF: 107/354, 30%). Despite significant differences in emergency admission, ambulance use, urinary catheter use on admission, and Barthel index on admission, the weighted cohort became well balanced for the recorded baseline variables.

**Table 1:** Baseline characteristics of patients with isolated C2 odontoid fracture before and after matching weight.

Characteristics	Unweighted cohort									Weighted cohort								
	Total		Halo-vest		ASF		PSF		*p*	Total		Halo-vest		ASF		PSF		*p*
	(n = 891)		(n = 463)		(n = 74)		(n = 354)			(n = 891)		(n = 423)		(n = 100)		(n = 369)		
Age, years	78 ± 7.5		78 ± 7.5		79 ± 7.1		78 ± 7.5		0.80	78 ± 7.5		78 ± 7.6		79 ± 7.1		78 ± 7.5		0.49
Age category, years									0.35									0.49
65–79	509	(57)	272	(59)	37	(50)	200	(57)		276	(58)	235	(56)	50	(50)	209	(57)	
≥ 80	382	(43)	191	(41)	37	(50)	154	(44)		203	(42)	188	(44)	50	(50)	160	(43)	
Male	366	(41)	184	(40)	29	(39)	153	(43)	0.57	382	(43)	181	(43)	39	(39)	162	(44)	0.68
Female	525	(59)	279	(60)	45	(61)	201	(57)		510	(57)	242	(57)	61	(61)	207	(56)	
BMI, kg/m^2^									0.15									0.99
Normal weight, 18.5–24.9	558	(71)	279	(60)	47	(64)	232	(66)		578	(71)	274	(75)	63	(64)	241	(65)	
Underweight, < 18.5	150	(15)	79	(17)	15	(20)	56	(16)		151	(13)	71	(11)	20	(20)	60	(16)	
Overweight, 25.0–29.9	94	(13)	50	(11)	7	(10)	37	(11)		92	(14)	44	(12)	9	(9.5)	39	(11)	
Obesity, ≥ 30.0	19	(1.5)	7	(1.5)	1	(1.4)	11	(3.1)		17	(1.8)	8	(1.8)	1	(1.4)	8	(2.1)	
Missing	70	(7.9)	48	(10.4)	4	(5.4)	18	(5.1)		53	(5.9)	26	(6.1)	5	(5.4)	22	(5.9)	
Smoking																		
Nonsmoker	622	(70)	338	(73)	50	(68)	234	(66)		606	(68)	295	(70)	67	(68)	244	(66)	
Smoker	163	(18)	75	(16)	16	(22)	72	(20)	0.23	174	(19)	80	(19)	22	(22)	72	(19)	0.78
Missing	106	(12)	50	(11)	8	(11)	48	(14)		111	(12)	47	(11)	11	(11)	53	(14)	
Academic hospital	752	(84)	392	(85)	63	(85)	297	(84)	0.94	757	(85)	359	(85)	85	(85)	313	(85)	0.99
Emergency admission	706	(79)	419	(91)	63	(85)	224	(63)	< 0.001	747	(84)	365	(86)	85	(85)	297	(81)	0.10
Ambulance use	426	(48)	251	(54)	37	(50)	138	(39)	< 0.001	434	(49)	209	(49)	50	(50)	175	(48)	0.84
Primary conditions																		
Admitted to ICU	15	(1.7)	9	(1.9)	3	(4.1)	3	(0.8)	0.12	16	(1.9)	8	(2.0)	4	(4.1)	4	(1.1)	0.15
Required oxygenation	98	(11)	52	(11)	10	(14)	36	(10)	0.69	111	(13)	53	(13)	13	(14)	45	(12)	0.97
Required hemodialysis	5	(0.6)	3	(0.6)	1	(1.4)	1	(0.3)	0.50	4	(0.5)	2	(0.5)	1	(1.4)	1	(0.4)	0.62
Required renal catheter	259	(29)	155	(34)	25	(34)	79	(22)	0.002	263	(30)	127	(30)	34	(34)	102	(28)	0.45
Comorbid conditions																		
Diabetes mellitus	143	(16)	73	(16)	11	(15)	59	(17)	0.90	145	(16)	67	(16)	15	(15)	63	(17)	0.84
Hypertension	256	(29)	131	(28)	23	(31)	102	(29)	0.89	264	(30)	125	(30)	31	(31)	108	(29)	0.95
Chronic lung disease	25	(2.8)	13	(2.8)	2	(2.7)	10	(2.8)	1.00	26	(2.9)	11	(2.7)	3	(2.7)	12	(3.2)	0.86
Cerebrovascular disease	64	(7.2)	39	(8.4)	4	(5.4)	21	(5.9)	0.32	57	(6.5)	27	(6.5)	5	(5.4)	25	(6.7)	0.82
Cardiac disease	131	(15)	58	(13)	12	(16)	61	(17)	0.16	141	(16)	65	(15)	16	(16)	60	(16)	0.94
Hepatic disease	80	(9.0)	44	(9.5)	7	(9.5)	29	(8.2)	0.80	78	(8.7)	36	(8.4)	9	(9.5)	33	(8.9)	0.97
Dementia	49	(5.5)	24	(5.2)	6	(8.1)	19	(5.4)	0.59	48	(5.4)	22	(5.3)	8	(8.1)	18	(4.9)	0.46
Osteoporosis	114	(13)	61	(13)	5	(6.8)	48	(14)	0.26	102	(11)	50	(12)	7	(6.8)	45	(12)	0.33
At least one comorbidity	526	(59)	280	(61)	44	(60)	202	(57)	0.61	525	(59)	248	(59)	59	(59)	218	(59)	0.99
JCS category									0.064									0.86
Alert	792	(89)	418	(90)	60	(81)	314	(89)		773	(87)	371	(88)	81	(81)	321	(87)	
Dizzy	99	(11)	45	(9.7)	14	(19)	40	(11)		118	(13)	52	(12)	19	(19)	47	(13)	
Barthel index on admission	5	(0–45)	5	(0–20)	5	(0–40)	5	(0–75)	0.002	5	(0–45)	5	(0–40)	5	(0–40)	5	(0–50)	0.066
CCI									0.19									0.45
≤ 1	708	(79)	378	(82)	59	(80)	271	(77)		710	(80)	339	(80)	79	(80)	292	(79)	
2	137	(15)	62	(13)	14	(19)	61	(17)		142	(16)	65	(15)	19	(19)	58	(16)	
≥ 3	46	(5.2)	23	(5.0)	1	(1.4)	22	(6.2)		18	(4.4)	20	(4.6)	1	(1.4)	18	(5.0)	

Data are presented as n (%), mean ± standard deviation, or median (interquartile range).

ASF, anterior spinal fixation; PSF, posterior spinal fixation; BMI, body mass index; ICU, intensive care unit; JCS, Japan Coma Scale; CCI, Charlson comorbidity index.

Table 2 shows the clinical outcomes among the treatments before and after weighting. Overall and 30-day in-hospital death occurred in 45 (5.1%) and 10 (1.1%) patients, respectively. The proportion of patients with at least one complication was 15%, and the most common complications after admission were respiratory complications (7.4%). The halo-vest group had a significantly longer PLOS than the ASF and PSF groups and significantly lower total costs than the PSF group. Univariable analysis in the weighted cohort showed that (i) in-hospital death was higher in the halo-vest group (6.6%) than the ASF (4.1%) and PSF (4.7%) groups with no significant difference, (ii) at least one complication was not significantly different among the treatments, and (iii) the PLOS was significantly longer in the halo-vest group than in the ASF or PSF group. Regarding complications after admission in the weighted cohort, the proportion of respiratory complications, cardiac events, and stroke were lower in the halo-vest group than in the ASF or PSF group.

**Table 2:** Clinical outcomes of patients before and after inverse probability treatment weighting.

Outcome	Unweighted cohort									Weighted cohort								
	Total		Halo-vest		ASF		PSF		*p*	Total		Halo-vest		ASF		PSF		*p*
	(n = 891)		(n = 463)		(n = 74)		(n = 354)			(n = 891)		(n = 423)		(n = 100)		(n = 368)		
Overall in-hospital death	45	(5.1)	26	(5.6)	3	(4.1)	16	(4.5)	0.72	49	(5.5)	28	(6.6)	4	(4.1)	17	(4.7)	0.37
30-day in-hospital death	10	(1.1)	5	(1.1)	1	(1.4)	4	(1.1)	0.98	10	(1.2)	6	(1.5)	1	(1.4)	3	(0.9)	0.71
At least one complication	132	(15)	60	(13)	14	(19)	58	(16)	0.23	137	(15)	57	(14)	19	(19)	61	(17)	0.28
Post-treatment length of stay, days	37	(20–70)	63	(29–88)	31	(21–54)	27	(17–42)	< 0.001	36	(20–68)	62	(28–88)	31	(21–54)	28	(18–46)	< 0.001
Total cost, thousand dollars	26 ± 13		24 ± 11		22 ± 13		29 ± 13		0.001	26 ± 13		23 ± 11		22 ± 13		31 ± 13		< 0.001
Complications																		
Sepsis	6	(0.7)	5	(1.1)	0	(0.0)	1	(0.3)	0.29	5	(0.6)	5	(1.2)	0	(0.0)	0	(0.1)	0.06
Pulmonary embolism	2	(0.2)	0	(0.0)	0	(0.0)	2	(0.6)	0.22	3	(0.5)	0	(0.0)	0	(0.0)	3	(0.7)	0.12
Respiratory complications	66	(7.4)	31	(6.7)	6	(8.1)	29	(8.2)	0.70	70	(7.8)	30	(7.0)	8	(8.1)	32	(8.6)	0.71
Cardiac events	17	(1.9)	5	(1.1)	3	(4.1)	9	(2.5)	0.12	20	(2.3)	5	(1.3)	4	(4.1)	11	(2.9)	0.11
Stroke	11	(1.2)	3	(0.6)	2	(2.7)	6	(1.7)	0.20	12	(1.4)	3	(0.8)	3	(2.7)	6	(1.7)	0.17
Urinary tract infection	25	(2.8)	14	(3.0)	1	(1.4)	10	(2.8)	0.72	23	(2.6)	11	(2.6)	1	(1.4)	11	(3.0)	0.55
Renal failure	7	(0.8)	2	(0.4)	0	(0.0)	5	(1.4)	0.21	7	(0.8)	2	(0.5)	0	(0.0)	5	(1.3)	0.24
Barthel index at discharge	85	(50–100)	85	(50–100)	80	(30–100)	85	(50–100)	0.65	80	(45–100)	85	(50–100)	80	(30–100)	80	(45–100)	0.011

Data are presented as n (%), mean ± standard deviation, or median (interquartile range).

ASF, anterior spinal fixation; PSF, posterior spinal fixation.

Table 3 shows the results of logistic regression and linear regression analyses of the main outcomes before and after matching weight. In the weighted cohort, there was no significant difference in overall in-hospital death between the halo-vest group and the ASF group (OR 0.60; 95% CI 0.17–2.07; *p* = 0.42) or PSF group (OR 0.70; 95% CI 0.35–1.44; *p* = 0.34). Although there was no significant difference in patients who developed at least one complication among the procedures, halo-vest immobilization was significantly associated with a longer PLOS than ASF (regression coefficient, − 25 days; 95% CI − 32.9 to − 17.5; *p* < 0.001) and PSF (regression coefficient, − 25 days; 95% CI − 29.6 to − 19.7; *p* < 0.001).

**Table 3:** Logistic regression and multiple linear regression models of main outcomes before and after weighting.

	Unweighted cohort			Weighted cohort		
Logistic regression model	Odds ratio	95% Confidence interval	*p*	Odds ratio	95% Confidence interval	*p*
Overall in-hospital death						
Halo-vest	Reference	–	–	Reference	–	–
ASF	0.71	0.21 to 2.41	0.58	0.60	0.17 to 2.07	0.42
PSF	0.80	0.42 to 1.51	0.48	0.70	0.35 to 1.44	0.34
At least one complication						
Halo-vest	Reference	–	–	Reference	–	–
ASF	1.57	0.82 to 3.00	0.17	1.49	0.77 to 2.90	0.24
PSF	1.32	0.89 to 1.95	0.17	1.26	0.80 to 1.99	0.31

Linear regression model	Regression coefficient (SE)	95% Confidence interval	*p*	Regression coefficient	95% Confidence interval	*P*
Post-treatment length of stay (days)						
Halo-vest	Reference	–	–	Reference	–	–
ASF	− 26	− 34.4 to − 17.1	< 0.001	− 25	− 32.9 to − 17.5	< 0.001
PSF	− 27	− 32.1 to − 22.4	< 0.001	− 25	− 29.6 to − 19.7	< 0.001

ASF, anterior spinal fixation; PSF, posterior spinal fixation; SE, standard error.

**Figure 2:**
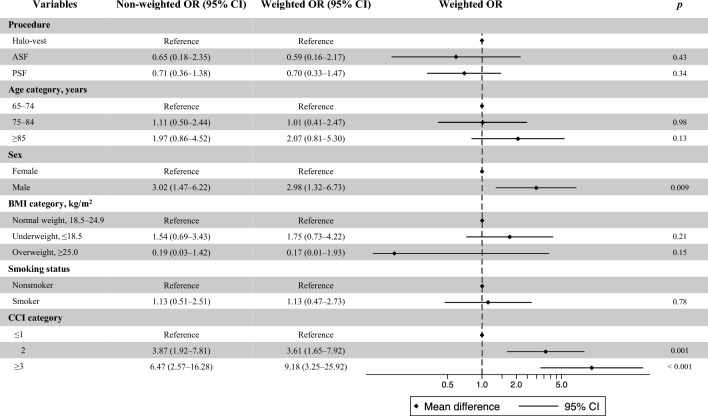
Risk factors for in-hospital death. Non-weighted and weighted ORs for each subgroup are shown. ORs and 95% CIs were obtained by multivariable logistic regression analysis adjusted by procedure, age category, sex, BMI category, smoking status, and CCI category. The square and horizontal bars represent the mean difference and 95% CI, respectively. OR, odds ratio; CI, confidence interval; ASF, anterior spinal fixation; PSF, posterior spinal fixation; BMI, body mass index; CCI, Charlson comorbidity index.

Figure 2 shows the results of the multivariable logistic regression analysis for in-hospital death before and after weighting. Male sex and a higher CCI category were independent risk factors for in-hospital mortality (male sex: OR 3.02; 95% CI 1.47–6.22; *p* = 0.003; CCI category 2: OR 3.61; 95% CI 1.65–7.92;* p* = 0.001; CCI of ≥ 3: OR 9.18; 95% CI 3.25–25.92; *p* < 0.001). The results of the sensitivity analyses were similar to those of the main analyses (Appendix Tables S1–S6).

## Discussion

We used a nationwide database and conducted a propensity score-based matching weight analysis to compare clinical outcomes of halo-vest immobilization, ASF, and PSF for elderly patients with isolated C2 odontoid fracture. In-hospital mortality and the development of at least one complication were not significantly different among the three procedures, whereas the PLOS was longer in the halo-vest group than in the surgery groups. Male sex and a higher CCI were independent risk factors for in-hospital mortality.

Halo-vest has been considered to be associated with higher mortality than surgical treatment in patients with C2 odontoid fracture, especially elderly patients^7,20^. Furthermore, in the latest meta-analysis, conservative treatment showed a trend toward higher mortality than surgical treatment^21^. The present study also showed relatively higher mortality in the halo-vest group than in the ASF or PSF group. However, halo-vest immobilization was not an independent risk factor for in-hospital death. One reason for higher mortality with conservative treatment may be selection bias due to limited settings of the target population. Most previous studies may have included critically ill patients with C2 fracture who could not be treated surgically. Furthermore, the sample sizes were small, even in the meta-analysis^1,2,7^. According to our results, the difference in in-hospital mortality between halo-vest immobilization and surgery may be slight. Halo-vest immobilization can be an option for C2 odontoid fracture if the patient cannot be treated surgically even when the fracture should be initially stabilized with surgery.

Respiratory complications are a cause of increased mortality of elderly patients who undergo halo-vest immobilization, and surgical treatment can reportedly decrease the incidence of pneumonia, cardiac arrest, and respiratory failure^6^. However, several studies showed no significant difference in complications between conservative and surgical treatment^6,8^. In the present study, complications including pneumonia, heart failure, and stroke were less common in the halo-vest group than in the ASF and PSF groups. Respiratory and cardiac complications can also occur as a result of surgery or general anesthesia, especially in elderly patients, who tend to have higher comorbidities and lower cardiac function^22^. Because surgical treatment may have more complications than halo-vest immobilization in elderly patients, careful attention is needed to avoid adverse events after surgical treatment of C2 odontoid fracture.

Optimal treatment for odontoid fracture has been discussed over the years. Previous studies have revealed that surgical treatment is more effective than conservative treatment for inducing bony fusion^23^. However, fibrous fusion is a more acceptable outcome than morbidity or mortality associated with surgery^23^. Thus, osseous union is not a prerequisite to obtaining satisfactory clinical outcomes in elderly patients. Additionally, the association between bony fusion and mortality remains inconsistent if neurological complications are absent^23^. In the present study, male sex and a higher CCI were strongly associated with in-hospital death in patients with isolated C2 odontoid fracture. Among elderly patients, pre-existing comorbidities themselves can be associated with mortality^24^. A comprehensive decision is necessary regardless of treatment type for C2 odontoid fracture, especially in terms of age, sex, and comorbidities.

This study has several limitations. First, we could not obtain data on the type of fracture, severity of instability, degree of dislocation, or surgical techniques details from the database. Second, despite using propensity score-based analysis, unmeasured confounding may not have been completely removed. The above-mentioned unavailable data may have been an unmeasured potential confounder affecting the indication for each treatment type. However, because more severe conditions make clinicians more likely to choose surgery, the surgery group likely had patients with more severe fractures. We conducted a sensitivity analyses, and the results were unchanged. Third, the database provides no data on outcomes after discharge. However, we assume that we covered most of the early adverse events because of the relatively long length of index hospitalization in Japan (median LOS for odontoid fracture is 31 days)^25^. Despite these limitations, we believe that our findings will have a significant impact on future treatment.

In conclusion, our study showed that the treatment type (halo-vest immobilization, ASF, or PSF) was not significantly associated with in-hospital mortality. Because elderly people are susceptible to higher comorbidity and baseline mortality rates, careful management may be required when these patients are male or have a higher CCI, regardless of treatment type for isolated C2 odontoid fracture.

### Supplementary Information

Supplementary Information.

## Data Availability

The datasets analyzed during the current study are not publicly available because of contracts with the hospitals providing data to the database but are partially available from the corresponding author on reasonable request.

## Code availability

All statistical analyses were performed with STATA/MP version 15 software (StataCorp, College Station, TX, USA). The codes are available to interested researchers upon request to the corresponding author.
