# Supplementary material for: Risk factors for early mortality in elderly patients with unstable isolated C2 odontoid fracture treated with halo-vest or surgery
Source: Sci Rep. 2023 Oct 20;13:17962. doi: 10.1038/s41598-023-45180-6 (PMC10589273; doi:10.1038/s41598-023-45180-6)
Supplement: Supplementary file 1 — Supplementary Information. [file 41598_2023_45180_MOESM1_ESM.docx]

Supplementary appendix. Sensitivity analyses for overall in-hospital mortality

Comparison of halo-vest immobilization with surgical procedures (both anterior spinal fixation and posterior spinal fixation)

Table of contents

Supplementary Tables 1–3: Propensity score-matching analysis for halo-vest immobilization and surgery groups

Supplementary Tables 4–6: Matching weight analysis between halo-vest immobilization and surgery groups

Supplementary Table 1. Baseline characteristics of patients with isolated C2 odontoid fracture before and after propensity score matching

Supplementary Table 2. Outcomes of patients with isolated C2 odontoid fracture after propensity score matching

Supplementary Table 3. Odds ratios for in-hospital death among patients with isolated C2 odontoid fracture after propensity score matching

Supplementary Table 4. Baseline characteristics of patients with isolated C2 odontoid fracture before and after matching weight

Supplementary Table 5. Outcomes of patients with isolated C2 fracture after matching weight

Supplementary Table 6. Odds ratios for in-hospital death among patients with isolated C2 odontoid fracture in matching cohort

Supplementary Tables S1–S3: Propensity score-matching analysis for halo-vest immobilization and surgery groups

Table S1

| Table S1. Baseline characteristics of patients with isolated C2 odontoid fracture before and after propensity score matching | | | | | | | | | | | | |
| --- | --- | --- | --- | --- | --- | --- | --- | --- | --- | --- | --- | --- |
| Characteristics | Unmatched cohort | | | | | | | Matched cohort | | | | |
|  | Total | | Halo-vest | | Surgery | | ASD, % | Halo-vest | | Surgery | | ASD, % |
|  | (n = 891) | | (n = 463) | | (n = 428) | |  | (n = 309) | | (n = 309) | |  |
| Age, years | 78 ± 7.5 | | 78 ± 7.5 | | 78 ± 7.5 | | 2.8 | 78 ± 7.9 | | 78 ± 7.3 | | 1.5 |
| Male | 366 | (41) | 184 | (40) | 182 | (43) | 5.7 | 138 | (45) | 139 | (45) | 0.6 |
| BMI, kg/m^2^ | 525 ± 59 | | 21 ± 3.9 | | 21 ± 3.7 | | 4.2 | 21 ± 3.9 | | 21 ± 3.5 | | 1.2 |
| BMI category, kg/m^2^ |  |  |  |  |  |  |  |  |  |  |  |  |
| Normal weight, 18.5–24.9 | 558 | (71) | 279 | (60) | 279 | (65) | 10 | 197 | (64) | 205 | (66) | 5.4 |
| Underweight, <18.5 | 150 | (15) | 79 | (17) | 71 | (17) | 1.3 | 56 | (18) | 48 | (16) | 6.9 |
| Overweight, 25.0–29.9 | 94 | (13) | 50 | (11) | 44 | (10) | 1.7 | 35 | (11) | 32 | (10) | 3.1 |
| Obesity, ≥30.0 | 19 | (1.5) | 7 | (1.5) | 12 | (2.8) | 8.9 | 6 | (1.9) | 5 | (1.6) | 2.4 |
| Missing | 70 | (7.9) | 48 | (10) | 22 | (5.1) | 20 | 15 | (4.9) | 19 | (6.1) | 5.7 |
| Smoking |  |  |  |  |  |  |  |  |  |  |  |  |
| Nonsmoker | 622 | (70) | 338 | (84) | 340 | (79) | 16 | 209 | (68) | 203 | (66) | 4.1 |
| Smoker | 163 | (18) | 75 | (16) | 88 | (21) | 11 | 55 | (18) | 59 | (19) | 3.3 |
| Missing | 106 | (12) | 50 | (11) | 56 | (13) | 7.1 | 45 | (15) | 47 | (15) | 1.8 |
| Academic hospital | 752 | (84) | 392 | (85) | 360 | (84) | 1.5 | 256 | (83) | 261 | (85) | 4.4 |
| Emergency admission | 706 | (79) | 419 | (91) | 287 | (67) | 60 | 265 | (86) | 276 | (89) | 11 |
| Ambulance use | 426 | (48) | 251 | (54) | 175 | (41) | 27 | 157 | (51) | 161 | (52) | 2.6 |
| Primary conditions |  |  |  |  |  |  |  |  |  |  |  |  |
| Admitted to ICU | 15 | (1.7) | 9 | (1.9) | 6 | (1.4) | 4.2 | 6 | (1.9) | 6 | (1.9) | 0.0 |
| Required oxygenation | 98 | (11) | 52 | (11) | 46 | (11) | 1.5 | 34 | (11) | 41 | (13) | 6.9 |
| Required hemodialysis | 5 | (0.6) | 3 | (0.6) | 2 | (0.5) | 2.4 | 1 | (0.3) | 2 | (0.6) | 4.6 |
| Required renal catheter | 259 | (29) | 155 | (34) | 104 | (24) | 20 | 94 | (30) | 96 | (31) | 1.4 |
| Comorbid conditions |  |  |  |  |  |  |  |  |  |  |  |  |
| Diabetes mellitus | 143 | (16) | 73 | (16) | 70 | (16) | 1.6 | 42 | (14) | 52 | (17) | 9.0 |
| Hypertension | 256 | (29) | 131 | (28) | 125 | (29) | 2.0 | 98 | (32) | 92 | (30) | 4.2 |
| Chronic lung disease | 25 | (2.8) | 13 | (2.8) | 12 | (2.8) | 0.0 | 8 | (2.6) | 8 | (2.6) | 0.0 |
| Cerebrovascular disease | 64 | (7.2) | 39 | (8.4) | 25 | (5.8) | 10 | 21 | (6.8) | 22 | (7.1) | 1.3 |
| Cardiac disease | 131 | (15) | 58 | (13) | 73 | (17) | 13 | 47 | (15) | 48 | (16) | 0.9 |
| Hepatic disease | 80 | (9.0) | 44 | (9.5) | 36 | (8.4) | 3.8 | 31 | (10) | 30 | (9.7) | 1.1 |
| Dementia | 49 | (5.5) | 24 | (5.2) | 25 | (5.8) | 2.9 | 19 | (6.1) | 17 | (5.5) | 2.8 |
| Osteoporosis | 114 | (13) | 61 | (13) | 53 | (12) | 2.4 | 33 | (11) | 37 | (12) | 4.1 |
| JCS category |  |  |  |  |  |  |  |  |  |  |  |  |
| Alert | 792 | (89) | 418 | (90) | 374 | (87) | 9.2 | 272 | (88) | 264 | (85) | 7.6 |
| Dizzy | 99 | (11) | 45 | (9.7) | 54 | (13) | 9.2 | 37 | (12) | 45 | (15) | 7.6 |
| Barthel index on admission | 5 | (0–45) | 5 | (0–20) | 5 | (0–60) | 29 | 5 | (0–35) | 5 | (0–30) | 3.0 |
| CCI |  |  |  |  |  |  |  |  |  |  |  |  |
| ≤1 | 708 | (79) | 378 | (82) | 330 | (77) | 11 | 250 | (81) | 244 | (79) | 4.8 |
| 2 | 137 | (15) | 62 | (13) | 75 | (18) | 12 | 46 | (15) | 52 | (17) | 5.3 |
| ≥3 | 46 | (5.2) | 23 | (5.0) | 23 | (5.4) | 1.8 | 13 | (4.2) | 13 | (4.2) | 0.0 |
| Data are presented as n (%), mean ± standard deviation, or median (interquartile range).  BMI, body mass index; ICU, intensive care unit; JCS, Japan Coma Scale; ASD, absolute standardized difference; CCI, Charlson comorbidity index | | | | | | | | | | | | |

Table S2

| Table S2. Outcomes of patients with isolated C2 odontoid fracture after propensity score matching | | | | | | | |
| --- | --- | --- | --- | --- | --- | --- | --- |
| Outcomes | Matched cohort | | | | | | |
|  | Total | | Halo-vest | | Surgery | | *p* |
|  | (n = 618) | | (n = 309) | | (n = 309) | |  |
| In-hospital death | 33 | (5.3) | 21 | (6.8) | 12 | (3.9) | 0.11 |
| 30-day death | 8 | (1.3) | 5 | (1.6) | 3 | (1.0) | 0.48 |
| At least one complication | 91 | (15) | 39 | (13) | 52 | (17) | 0.14 |
| Post-treatment length of stay, days | 38 | (21–72) | 63 | (28–88) | 29 | (18–47) | <0.001 |
| Total cost, thousand dollars | 26 ± 13 | | 23 ± 11 | | 29 ± 13 | | <0.001 |
| Complications |  |  |  |  |  |  |  |
| Sepsis | 3 | (0.5) | 3 | (1.0) | 0 | (0.0) | 0.083 |
| Pulmonary embolism | 2 | (0.3) | 0 | (0.0) | 2 | (0.6) | 0.16 |
| Respiratory complications | 46 | (7.4) | 21 | (6.8) | 25 | (8.1) | 0.54 |
| Cardiac events | 14 | (2.3) | 5 | (1.6) | 9 | (2.9) | 0.28 |
| Stroke | 9 | (1.5) | 2 | (0.6) | 7 | (2.3) | 0.093 |
| Urinary tract infection | 15 | (2.4) | 7 | (2.3) | 8 | (2.6) | 0.79 |
| Renal failure | 4 | (0.7) | 2 | (0.6) | 2 | (0.6) | 1.0 |
| Barthel index at discharge | 85 | (45–100) | 83 | (50–100) | 80 | (45–100) | 0.96 |
| Data are presented as n (%), mean ± standard deviation, or median (interquartile range). | | | | | | | |

| Table S3. Odds ratios for in-hospital death among patients with isolated C2 odontoid fracture after propensity score matching | | | |
| --- | --- | --- | --- |
| Variables | Odds ratio | 95% Confidence interval | *p* |
| Procedures |  |  |  |
| Halo-vest | Reference | – | – |
| Surgery | 0.53 | 0.24 to 1.15 | 0.11 |
| Age, years |  |  |  |
| 65–74 | Reference | – | – |
| 75–84 | 1.00 | 0.37 to 2.71 | 1.00 |
| ≥85 | 2.44 | 0.90 to 6.64 | 0.08 |
| Male | 3.13 | 1.30 to 7.56 | 0.01 |
| BMI, kg/m^2^ |  |  |  |
| Normal weight, 18.5–24.9 | Reference |  |  |
| Underweight, <18.5 | 2.30 | 0.92 to 5.71 | 0.07 |
| Overweight, ≥25.0 | 0.16 | 0.16 to 1.35 | 0.22 |
| Missing | 0.80 | 0.09 to 6.89 | 0.84 |
| Smoking status |  |  |  |
| Nonsmoker | Reference |  |  |
| Smoker | 0.91 | 0.34 to 2.42 | 0.85 |
| Missing | 0.62 | 0.19 to 2.03 | 0.43 |
| CCI |  |  |  |
| ≤1 | Reference |  |  |
| 2 | 4.01 | 1.75 to 9.46 | 0.001 |
| ≥3 | 12.8 | 4.26 to 38.76 | <0.001 |
| BMI, body mass index; CCI, Charlson comorbidity index | | | |

Table S3

Supplementary Tables S4–S6: Matching weight analysis between halo-vest immobilization and surgery groups

Table S4

| Table S4. Baseline characteristics of patients with isolated C2 odontoid fracture before and after matching weight | | | | | | | | | | | | |
| --- | --- | --- | --- | --- | --- | --- | --- | --- | --- | --- | --- | --- |
| Characteristics | Unweighted cohort | | | | | | | Weighted cohort | | | | |
|  | Total | | Halo-vest | | Surgery | | ASD, % | Halo-vest | | Surgery | | ASD, % |
|  | (n = 891) | | (n = 463) | | (n = 428) | |  | (n = 445) | | (n = 446) | |  |
| Age, years | 78 ± 7.5 | | 78 ± 7.5 | | 78 ± 7.5 | | 2.8 | 78 ± 7.5 | | 78 ± 7.3 | | 0.6 |
| Male | 366 | (41) | 184 | (40) | 182 | (43) | 5.7 | 191 | (43) | 190 | (43) | 0.5 |
| BMI, kg/m^2^ | 525 ± 59 | | 21 ± 3.9 | | 21 ± 3.7 | | 4.2 | 21 ± 3.3 | | 21 ± 3.2 | | 0.1 |
| BMI category, kg/m^2^ |  |  |  |  |  |  |  |  |  |  |  |  |
| Normal weight, 18.5–24.9 | 558 | (71) | 279 | (60) | 279 | (65) | 10 | 292 | (66) | 294 | (66) | 0.7 |
| Underweight, <18.5 | 150 | (15) | 79 | (17) | 71 | (17) | 1.3 | 74 | (17) | 73 | (16) | 0.8 |
| Overweight, 25.0–29.9 | 94 | (13) | 50 | (11) | 44 | (10) | 1.7 | 45 | (10) | 45 | (10) | 0.0 |
| Obesity, ≥30.0 | 19 | (1.5) | 7 | (1.5) | 12 | (2.8) | 8.9 | 8 | (1.8) | 8 | (1.7) | 0.8 |
| Missing | 70 | (7.9) | 48 | (10) | 22 | (5.1) | 20 | 26 | (5.9) | 27 | (5.9) | 0.3 |
| Smoking |  |  |  |  |  |  |  |  |  |  |  |  |
| Nonsmoker | 622 | (70) | 338 | (84) | 340 | (79) | 16 | 300 | (68) | 301 | (68) | 0.1 |
| Smoker | 163 | (18) | 75 | (16) | 88 | (21) | 11 | 83 | (19) | 84 | (19) | 0.3 |
| Missing | 106 | (12) | 50 | (11) | 56 | (13) | 7.1 | 62 | (14) | 61 | (14) | 0.6 |
| Academic hospital | 752 | (84) | 392 | (85) | 360 | (84) | 1.5 | 374 | (84) | 375 | (84) | 0.4 |
| Emergency admission | 706 | (79) | 419 | (91) | 287 | (67) | 60 | 382 | (86) | 385 | (86) | 1.1 |
| Ambulance use | 426 | (48) | 251 | (54) | 175 | (41) | 27 | 219 | (49) | 224 | (50) | 1.8 |
| Primary conditions |  |  |  |  |  |  |  |  |  |  |  |  |
| Admitted to ICU | 15 | (1.7) | 9 | (1.9) | 6 | (1.4) | 4.2 | 8 | (1.8) | 8 | (1.7) | 0.6 |
| Required oxygenation | 98 | (11) | 52 | (11) | 46 | (11) | 1.5 | 54 | (12) | 55 | (12) | 0.6 |
| Required hemodialysis | 5 | (0.6) | 3 | (0.6) | 2 | (0.5) | 2.4 | 2 | (0.6) | 2 | (0.5) | 0.7 |
| Required renal catheter | 259 | (29) | 155 | (34) | 104 | (24) | 20 | 134 | (30) | 135 | (30) | 0.2 |
| Comorbid conditions |  |  |  |  |  |  |  |  |  |  |  |  |
| Diabetes mellitus | 143 | (16) | 73 | (16) | 70 | (16) | 1.6 | 71 | (16) | 75 | (17) | 2.3 |
| Hypertension | 256 | (29) | 131 | (28) | 125 | (29) | 2.0 | 131 | (29) | 129 | (29) | 1.0 |
| Chronic lung disease | 25 | (2.8) | 13 | (2.8) | 12 | (2.8) | 0.0 | 12 | (2.8) | 13 | (2.9) | 0.9 |
| Cerebrovascular disease | 64 | (7.2) | 39 | (8.4) | 25 | (5.8) | 10 | 30 | (6.7) | 30 | (6.8) | 0.3 |
| Cardiac disease | 131 | (15) | 58 | (13) | 73 | (17) | 13 | 67 | (15) | 68 | (15) | 0.2 |
| Hepatic disease | 80 | (9.0) | 44 | (9.5) | 36 | (8.4) | 3.8 | 38 | (8.5) | 40 | (9.1) | 1.9 |
| Dementia | 49 | (5.5) | 24 | (5.2) | 25 | (5.8) | 2.9 | 24 | (5.5) | 23 | (5.1) | 1.7 |
| Osteoporosis | 114 | (13) | 61 | (13) | 53 | (12) | 2.4 | 53 | (12) | 52 | (12) | 0.4 |
| JCS category |  |  |  |  |  |  |  |  |  |  |  |  |
| Alert | 792 | (89) | 418 | (90) | 374 | (87) | 9.2 | 392 | (88) | 392 | (88) | 1.2 |
| Dizzy | 99 | (11) | 45 | (9.7) | 54 | (13) | 9.2 | 47 | (10) | 54 | (12) | 1.2 |
| Barthel index on admission | 5 | (0–45) | 5 | (0–20) | 5 | (0–60) | 29 | 5 | (0–45) | 5 | (0–45) | 2.3 |
| CCI |  |  |  |  |  |  |  |  |  |  |  |  |
| ≤1 | 708 | (79) | 378 | (82) | 330 | (77) | 11 | 355 | (80) | 357 | (80) | 0.6 |
| 2 | 137 | (15) | 62 | (13) | 75 | (18) | 12 | 67 | (15) | 67 | (15) | 0.4 |
| ≥3 | 46 | (5.2) | 23 | (5.0) | 23 | (5.4) | 1.8 | 22 | (5.0) | 22 | (5.0) | 0.3 |
| Data are presented as n (%), mean ± standard deviation, or median (interquartile range).  BMI, body mass index; ICU, intensive care unit; JCS, Japan Coma Scale; ASD, absolute standardized difference; IQR, interquartile range; CCI, Charlson comorbidity index | | | | | | | | | | | | |

Table S5

| Table S5. Outcomes of patients with isolated C2 odontoid fracture after matching weight | | | | | | | |
| --- | --- | --- | --- | --- | --- | --- | --- |
| Outcomes | Matched cohort | | | | | | |
|  | Total | | Halo-vest | | Surgery | | *p* |
|  | (n = 891) | | (n = 445) | | (n = 446) | |  |
| In-hospital death | 48 | (5.4) | 30 | (6.8) | 18 | (4.1) | 0.08 |
| 30-day death | 11 | (1.3) | 7 | (1.5) | 4 | (1.0) | 0.38 |
| At least one complication | 135 | (15) | 60 | (14) | 75 | (17) | 0.226 |
| Post-treatment length of stay, days | 38 | (21–72) | 62 | (28–88) | 29 | (18–47) | <0.001 |
| Total cost, thousand dollars | 26 ± 13 | | 23 ± 11 | | 29 ± 13 | | <0.001 |
| Complications |  |  |  |  |  |  |  |
| Sepsis | 5 | (0.6) | 5 | (1.1) | 0 | (0.0) | 0.031 |
| Pulmonary embolism | 3 | (0.3) | 0 | (0.0) | 3 | (0.7) | 0.25 |
| Respiratory complications | 67 | (7.6) | 31 | (7.0) | 36 | (8.1) | 0.61 |
| Cardiac events | 18 | (2.4) | 6 | (1.4) | 12 | (2.7) | 0.23 |
| Stroke | 11 | (1.3) | 3 | (0.8) | 8 | (1.9) | 0.22 |
| Urinary tract infection | 15 | (2.4) | 11 | (2.5) | 11 | (2.6) | 1.00 |
| Renal failure | 5 | (0.8) | 3 | (0.6) | 4 | (0.9) | 1.00 |
| Barthel index at discharge | 85 | (50–100) | 85 | (50–100) | 80 | (45–100) | 0.85 |
| Data are presented as n (%), mean ± standard deviation, or median (interquartile range). | | | | | | | |

| Table S6. Odds ratios for in-hospital death among patients with isolated C2 odontoid fracture in matching cohort | | | |
| --- | --- | --- | --- |
| Variables | Odds ratio | 95% Confidence interval | *p* |
| Procedure |  |  |  |
| Halo-vest | Reference | – | – |
| Surgery | 0.60 | 0.28 to 1.27 | 0.18 |
| Age, years |  |  |  |
| 65–74 | Reference | – | – |
| 75–84 | 1.05 | 0.41 to 2.72 | 0.92 |
| ≥85 | 2.12 | 0.78 to 5.74 | 0.14 |
| Male | 3.21 | 1.35 to 7.64 | 0.009 |
| BMI, kg/m^2^ |  |  |  |
| Normal weight, 18.5–24.9 | Reference |  |  |
| Underweight, <18.5 | 2.23 | 0.90 to 5.51 | 0.08 |
| Overweight, ≥25.0 | 0.20 | 0.01 to 3.07 | 0.25 |
| Missing | 0.62 | 0.08 to 4.79 | 0.64 |
| Smoking status |  |  |  |
| Nonsmoker | Reference |  |  |
| Smoker | 1.03 | 0.40 to 2.64 | 0.91 |
| Missing | 0.71 | 0.22 to 2.33 | 0.57 |
| CCI |  |  |  |
| ≤1 | Reference |  |  |
| 2 | 3.80 | 1.63 to 8.84 | 0.002 |
| ≥3 | 9.06 | 3.15 to 26.03 | <0.001 |
| BMI, body mass index; CCI, Charlson comorbidity index | | | |

Table S6
